# Editorial: Biomarkers in allergic eczema

**DOI:** 10.3389/falgy.2025.1749379

**Published:** 2025-12-03

**Authors:** Anish Raj Maskey, Pranay Bharadwaj, Jan Geliebter

**Affiliations:** 1 Department of Pathology, Microbiology & Immunology, New York Medical College, Valhalla, NY, United States; 2 Department of Microbiology & Immunology, Touro College of Osteopathic Medicine, Great Falls, MT, United States; 3 Biotherapeutic Discovery, Boehringer Ingelheim Pharmaceuticals Inc., Ridgefield, CT, United States

**Keywords:** biomarker, eczema, personalized therapy, Th2 (type-2) immune responses, endotype

Eczema is a complex, heterogeneous inflammatory disease of the skin. Patients exhibit diverse immunopathological mechanisms and treatment responses, indicating the existence of multiple disease endotypes. Current therapies fail to achieve sustained disease control across all patient subgroups. Therefore, the identification of reliable biomarkers is essential for monitoring disease progression and therapeutic outcomes. This Research Topic- *Biomarkers in Allergic Eczema*- aims to elucidate the underlying immunological mechanisms, discover diagnostic, prognostic, and predictive biomarkers, and define their utility in patient stratification and assessment of treatment response. The development of clinically useful, cost-efficient, and non-invasive biomarkers for allergic eczema would greatly enhance personalized management strategies. These types of advances could transform clinical practice by enabling the early detection and accurate classification of disease endotypes, along with real-time monitoring of therapeutic responses.

Sargen et al. identified emerging diagnostic and prognostic biomarkers in allergic contact dermatitis (ACD), including *ADAM8, CD47, IL-37,* and *loricrin*. Sasaki et al. proposed neuroimmune mediators such as *IL-31* and *TRPV1* as therapeutic targets in ACD. Together, these reviews emphasize the importance of integrating skin and blood biomarker networks, multi-omics, and longitudinal cohort validation to advance the personalized management of allergic and inflammatory skin diseases in patients of all ages. Maskey et al. reported topical steroid withdrawal (TSW) as an emerging inflammatory syndrome following chronic corticosteroid use. They discussed the mechanisms involving nitric oxide–mediated vasodilation, epidermal cortisol dysregulation, microbiome imbalance, and cytokine rebound as prime drivers of TSW. The authors advocate clinical recognition, diagnostic refinement, and multidisciplinary management, including gradual tapering, skin barrier repair, and mental health support to reduce the occurrence of TSW. Song et al. explored the use of systemic inflammatory burden as a biomarker of eczema in 3,397 children and adolescents using the NHANES database. This study introduced the systemic inflammatory response index (SIRI**)** as a promising, easily measurable biomarker for systemic immune balance in allergic skin diseases. The authors demonstrated that higher SIRI levels are inversely associated with eczema prevalence, suggesting a potential protective immunological balance. They also proposed that elevated SIRI levels may reflect stronger Th1 or regulatory *T* cell activity, which counteracts Th2-driven inflammation.

In a perspective article, Kataoka discussed the importance of biomarkers in personalizing healthcare to enable more effective treatment plan development in clinics, citing CCL17 as a key biomarker for atopic dermatitis and emphasizing its importance in the detection of subclinical manifestations of the disease. Early identification of subclinical inflammation enables proactive topical therapy and reduces unnecessary escalation to costly biologics or JAK inhibitors. Incorporating these biomarkers into precision medicine strategies can thus assist with improved disease control, cost-effective treatment options, and positive patient outcomes in an evolving era of advanced AD therapies. In another study, Chong et al. evaluated the clinical utility of strawberry- and tomato-specific IgE (S/T IgE) testing in 814 pediatric allergy cases, and found that only 2.2% (strawberry) and 5.4% (tomato) of tests aligned with clinically relevant outcomes, with severe allergy being rare. Conversely, the research group observed that exposure and reaction history (ERH) is a far stronger predictor of true allergy in comparison to systemic S/T IgE levels, offering high negative and low positive predictive value. These findings suggest that S/T IgE testing should be reserved for patients with a clear ERH, thereby reinforcing the importance of clinical history as the primary tool in diagnosis. Lazor et al. reviewed and outlined the current standards and emerging therapies for managing allergic eczema, ranging from emollients and topical corticosteroids to calcineurin inhibitors and systemic agents for severe cases, along with novel biologic therapeutic interventions, such as dupilumab and JAK inhibitors, which offer targeted and effective therapy. The multitude of therapeutic options in terms of both range and effectiveness, along with patient stratification, allows for personalized, proactive strategies and biomarker-driven approaches that reduce the financial and emotional burden of the disease on patients and their caregivers.

To investigate the function of mast cells in an *in situ* environment, Villanueva et al. investigated the use of intact human skin explant tissue to study human mast cell activation. Their Methods study describes the development and application of this full-thickness human skin specimen model. Spontaneous histamine release was investigated, along with induced FceR1 and MRGPRX2 activation. The use of acalabrutinib, QWF, and pertussis toxin to inhibit histamine release contributed to the verification of normal mast cell function. The use of “human skin punch biopsy discards” opens the way for *in situ* investigations of mast cell function and therapeutic inhibition.

Intake, metabolism, and use of vitamin B6 are important for brain function and the immune system. However, the role of vitamin B6 and its metabolites in eczema is understudied. Du and Li utilized data from the 2005–2006 National Health and Nutrition Examination Surveys (NHANES) to select 247 children and adolescents (aged 6–17) with eczema and 2009 without eczema. The active form of vitamin B6 (PLP), its inactive metabolite (4-PA), and the 4-PA/PLP ratio in serum were measured to determine their association with eczema. High levels of 4-PA and 4-PA/PLP were associated with eczema. No association was observed between dietary intake of vitamin B6 or serum PLP levels and eczema. These results suggest that the inclusion of vitamin B6 status in the screening of children may be of value for monitoring and perhaps predicting eczema.

In the future, integrating skin and blood biomarker networks, multi-omics, and longitudinal cohort validation will be pivotal in advancing the personalized management of allergic and inflammatory skin diseases across different age groups.

